# Integrated longitudinal multiomics study identifies immune programs associated with acute COVID-19 severity and mortality

**DOI:** 10.1172/JCI176640

**Published:** 2024-05-01

**Authors:** Jeremy P. Gygi, Cole Maguire, Ravi K. Patel, Pramod Shinde, Anna Konstorum, Casey P. Shannon, Leqi Xu, Annmarie Hoch, Naresh Doni Jayavelu, Elias K. Haddad, IMPACC Network, Elaine F. Reed, Monica Kraft, Grace A. McComsey, Jordan P. Metcalf, Al Ozonoff, Denise Esserman, Charles B. Cairns, Nadine Rouphael, Steven E. Bosinger, Seunghee Kim-Schulze, Florian Krammer, Lindsey B. Rosen, Harm van Bakel, Michael Wilson, Walter L. Eckalbar, Holden T. Maecker, Charles R. Langelier, Hanno Steen, Matthew C. Altman, Ruth R. Montgomery, Ofer Levy, Esther Melamed, Bali Pulendran, Joann Diray-Arce, Kinga K. Smolen, Gabriela K. Fragiadakis, Patrice M. Becker, Rafick P. Sekaly, Lauren I.R. Ehrlich, Slim Fourati, Bjoern Peters, Steven H. Kleinstein, Leying Guan

**Affiliations:** 1 Yale School of Medicine, New Haven, Connecticut, USA.; 2 The University of Texas at Austin, Austin, Texas, USA.; 3 UCSF, San Francisco, California, USA.; 4 La Jolla Institute for Immunology, La Jolla, California, USA.; 5 Centre for Heart Lung Innovation, University of British Columbia, Vancouver, Canada.; 6 Prevention of Organ Failure (PROOF) Centre of Excellence, Providence Research, Vancouver, British Columbia, Canada.; 7 Yale School of Public Health, New Haven, Connecticut, USA.; 8 Clinical and Data Coordinating Center (CDCC) and; 9 Precision Vaccines Program, Boston Children’s Hospital and Harvard Medical School, Boston, Massachusetts, USA.; 10 Benaroya Research Institute, Seattle, Washington, USA.; 11 Drexel University, Tower Health Hospital, Philadelphia, Pennsylvania, USA.; 12 The Immunophenotyping Assessment in a COVID-19 Cohort (IMPACC) Network is detailed in Supplemental Acknowledgments.; 13 David Geffen School of Medicine at the UCLA, Los Angeles, California, USA.; 14 Icahn School of Medicine at Mount Sinai, New York, New York, USA.; 15 Case Western Reserve University and University Hospitals of Cleveland, Cleveland, Ohio, USA.; 16 Oklahoma University Health Sciences Center, Oklahoma City, Oklahoma, USA.; 17 Broad Institute of MIT and Harvard, Cambridge, Massachusetts, USA.; 18 Department of Pediatrics, Boston Children’s Hospital and Harvard Medical School, Boston, Massachusetts, USA.; 19 Emory School of Medicine, Atlanta, Georgia, USA.; 20 Ignaz Semmelweis Institute, Interuniversity Institute for Infection Research, Medical University of Vienna, Vienna, Austria.; 21 National Institute of Allergy and Infectious Diseases (NIAID), NIH, Bethesda, Maryland, USA.; 22 Stanford University School of Medicine, Palo Alto, California, USA.; 23 Department of Pathology, Boston Children’s Hospital and Harvard Medical School, Boston, Massachusetts, USA.; 24 Department of Medicine, UCSD, La Jolla, California, USA.

**Keywords:** COVID-19, Immunology, Adaptive immunity, Innate immunity

## Abstract

**BACKGROUND:**

Patients hospitalized for COVID-19 exhibit diverse clinical outcomes, with outcomes for some individuals diverging over time even though their initial disease severity appears similar to that of other patients. A systematic evaluation of molecular and cellular profiles over the full disease course can link immune programs and their coordination with progression heterogeneity.

**METHODS:**

We performed deep immunophenotyping and conducted longitudinal multiomics modeling, integrating 10 assays for 1,152 Immunophenotyping Assessment in a COVID-19 Cohort (IMPACC) study participants and identifying several immune cascades that were significant drivers of differential clinical outcomes.

**RESULTS:**

Increasing disease severity was driven by a temporal pattern that began with the early upregulation of immunosuppressive metabolites and then elevated levels of inflammatory cytokines, signatures of coagulation, formation of neutrophil extracellular traps, and T cell functional dysregulation. A second immune cascade, predictive of 28-day mortality among critically ill patients, was characterized by reduced total plasma Igs and B cells and dysregulated IFN responsiveness. We demonstrated that the balance disruption between IFN-stimulated genes and IFN inhibitors is a crucial biomarker of COVID-19 mortality, potentially contributing to failure of viral clearance in patients with fatal illness.

**CONCLUSION:**

Our longitudinal multiomics profiling study revealed temporal coordination across diverse omics that potentially explain the disease progression, providing insights that can inform the targeted development of therapies for patients hospitalized with COVID-19, especially those who are critically ill.

**TRIAL REGISTRATION:**

ClinicalTrials.gov NCT04378777.

**FUNDING:**

NIH (5R01AI135803-03, 5U19AI118608-04, 5U19AI128910-04, 4U19AI090023-11, 4U19AI118610-06, R01AI145835-01A1S1, 5U19AI062629-17, 5U19AI057229-17, 5U19AI125357-05, 5U19AI128913-03, 3U19AI077439-13, 5U54AI142766-03, 5R01AI104870-07, 3U19AI089992-09, 3U19AI128913-03, and 5T32DA018926-18); NIAID, NIH (3U19AI1289130, U19AI128913-04S1, and R01AI122220); and National Science Foundation (DMS2310836).

## Abstract 2

Acute longitudinal multi-omic immune profiles from 1,152 participants hospitalized with SARS-CoV-2 reveal immune patterns involved with disease progression and inform the targeted development of therapies.

## Graphical abstract

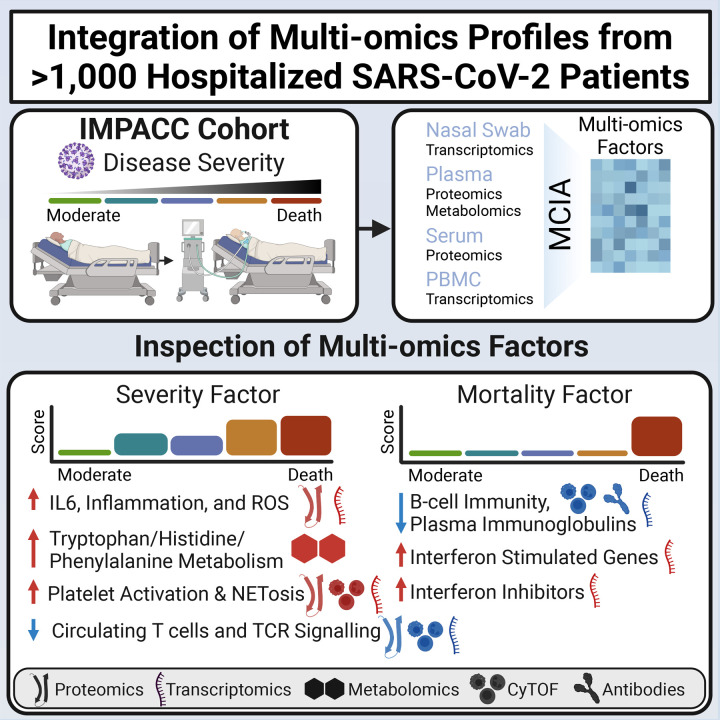

## Introduction

COVID-19, caused by SARS-CoV-2 infection, reflects a complex balance between viral replication, host immune response, and physiological manifestations such as hypoxia, organ dysfunction, and systemic inflammation (1–4). Patients hospitalized with COVID-19 exhibit a broad range of clinical outcomes, from moderate severity and a short hospital stay to critical illness with prolonged hospitalization and even death (5, 6). Profiling the immune response in a clinically diverse cohort would enable the linking of molecular and cellular mechanisms with these differential outcomes and could inform the development of targeted therapies.

Several hallmarks of severe COVID-19 have been identified, including overproduction of proinflammatory cytokines (7–9), lymphopenia (7, 10, 11), formation of neutrophil extracellular traps (NETs) (12), impaired IFN signaling (13–16), presence of anti-IFN autoantibodies (17), and immune senescence (18). Although these studies have identified many individual components underlying the pathophysiology of SARS-CoV-2 infection, it is still unclear how their temporal coordination interplay contributes to the observed heterogeneity of responses among hospitalized patients, especially among the critically ill. Why do some patients with COVID-19 survive, while others experience fatal illnesses despite apparently similar severity at hospital admission? Additionally, most existing studies focus on measurements from peripheral blood and leverage only one or a small number of assays, thus limiting opportunities to identify biologically relevant connections between tissues and mechanisms that operate across scales. Such a systems-level understanding requires longitudinal multiomics studies on large-scale and clinically well-defined hospitalized cohorts to characterize innate, adaptive, and mucosal immune cascades associated with disease severity (19–22).

The Immunophenotyping Assessment in a COVID-19 Cohort (IMPACC) study aims to gain a panoramic understanding of SARS-CoV-2 infection via the collection and analysis of detailed clinical, laboratory, and radiographic data along with longitudinal biologic samplings of blood and respiratory secretions from participants hospitalized with COVID-19 across the United States (23). Previously, IMPACC conducted deep immunophenotyping of longitudinal samples for 539 hospitalized participants mostly enrolled before September 2020 to identify biological states associated with SARS-CoV-2 disease course trajectories (6).

In the current work, we first carried out an integrative multiomics analysis of the existing IMPACC data to develop models that focused on 2 tasks: predicting disease severity and mortality. To test these models, we then generated independent immunophenotyping data on an additional 613 hospitalized IMPACC participants enrolled after September 2020. Our integrative analysis revealed strong orchestrated variations across serum soluble proteins (cytokines, chemokines, and secreted receptors), plasma proteins and metabolites, gene expression in PBMC and nasal swab samples, circulating immune cell frequencies, as well as viral loads and SARS-CoV-2 serum antibodies. For example, early dysregulation of metabolism in plasma, depressed B cell functions, and a disrupted balance in IFN signaling could distinguish fatality among critically ill patients whose disease severity levels were similar at hospital admission. Although the dysregulation of IFN signaling in severe COVID-19 has been hypothesized previously (24, 25), our integrative analysis revealed evidence for the potentially critical role of inhibitory genes in this dysregulation through investigation of the temporal coordination. In summary, we present a set of robust immunological findings that are capable of distinguishing between varying severities of COVID-19 among hospitalized patients and yield insight into the pathophysiology of severe disease. Our analysis strategy can be generalized to mine additional coordinated and temporal dynamics to investigate other infectious diseases beyond the current pandemic.

## Results

### Longitudinal multiomics profiling of SARS-CoV-2 infection response.

**Figure 1:**
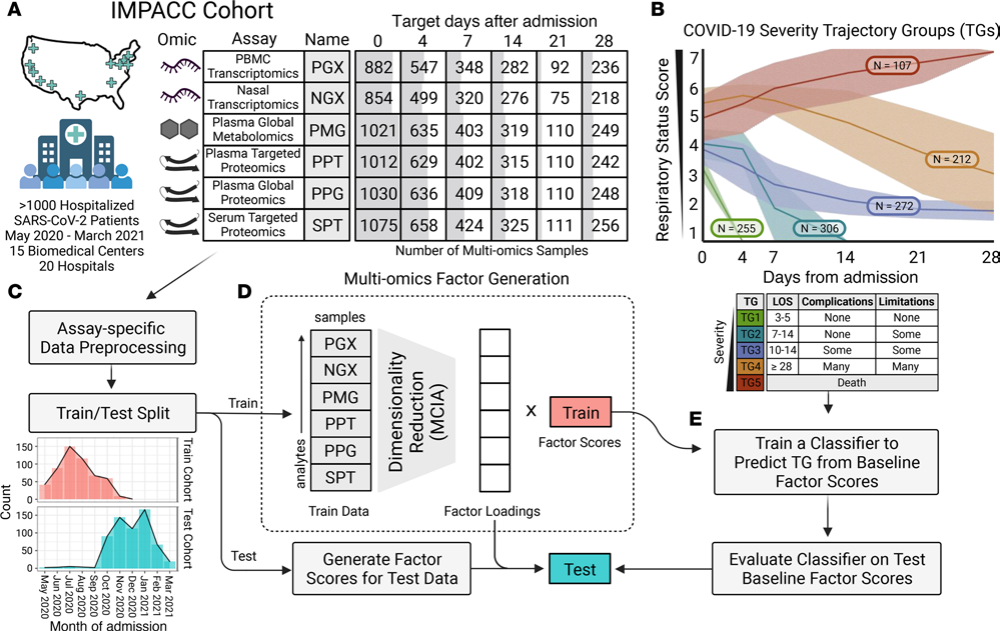
Data overview and multiomics factor generation. (**A**) Table with the number of samples used in the integrative analysis separated by assay (rows) and scheduled time of collection (columns). Cells are shaded to reflect the relative number of multiomics samples available. (**B**) Plot of clinical TG assignments for all IMPACC cohort participants (*n* = 1,164) and clinical descriptions for each TG. The *x* axis represents days from hospital admission, and the *y* axis represents the ordinal respiratory status score (1, 2 = discharged, 3–6 = hospitalized, 7 = fatal). The shaded region denotes the IQR of each TG. (**C**) Preprocessed data for different assays were split into training and test cohorts. (**D**) Dimensionality reduction was performed via MCIA on the training cohort assays to construct multiomics factors and loadings. (**E**) Baseline factor scores were used to train a classifier for predicting the TG with the model selected via cross-validation. The classifier was used to predict the TG for the testing cohort factor scores. This figure was created with BioRender.com.

The IMPACC study enrolled 1,152 participants from 20 US sites from May 2020 through March 2021, prior to the widespread rollout of SARS-CoV-2 vaccines (23). All participants were assigned to 1 of 5 COVID-19 trajectory groups (TGs) using latent class modeling based on a modified WHO score, referred to as the respiratory ordinal status (5) (Figure 1B). Groups ranged from TG1 (length of hospital stay [LOS] of ~3–5 days and with a largely uncomplicated hospital course); TG2 (LOS of ~7–14 days and discharged with no limitations); TG3 (LOS of ~10–14 days and discharged with limitations); TG4 (LOS of ~28 days or more), to TG5 (fatal illness by day 28). Patients win both TG4 and TG5 were considered critically ill, with TG5 uniquely containing participants who had fatal illness within the first 28 days of hospitalization.

We carried out and reported deep immunophenotyping for 539 IMPACC participants who were enrolled primarily before September 2020 (6). These data included profiling of targeted proteomics of serum (SPT), global plasma metabolomics (PMG), global and targeted proteomics profiles of plasma (PPG and PPT, respectively), as well as transcriptomics from PBMCs and nasal swabs (denoted as PGX and NGX, respectively). All assays were collectively measured longitudinally across 6 scheduled visits from day 0 (referred to as baseline) to 28 days after hospital admission (Figure 1A). In this work, we used these published profiles as training data to construct covarying immune programs and models that predict disease severity and mortality. We examined molecular immune programs using hold-out assays including those measuring immunological outcomes via serum antibody titers, virological outcomes via nasal viral loads, and whole blood cell frequencies via mass cytometry by time of flight (CyTOF). To evaluate the performance of the prediction models on independent data, we performed new deep immunophenotyping for an additional 613 IMPACC participants who were enrolled after September 2020 (Supplemental Table 1; supplemental material available online with this article; https://doi.org/10.1172/JCI176640DS1). In total, these IMPACC data included 20,544 distinct assay measurements comprising 3,077 multiomics profiles (referred to hereafter as samples).

### Multiomics factors predict clinical TGs.

**Figure 2:**
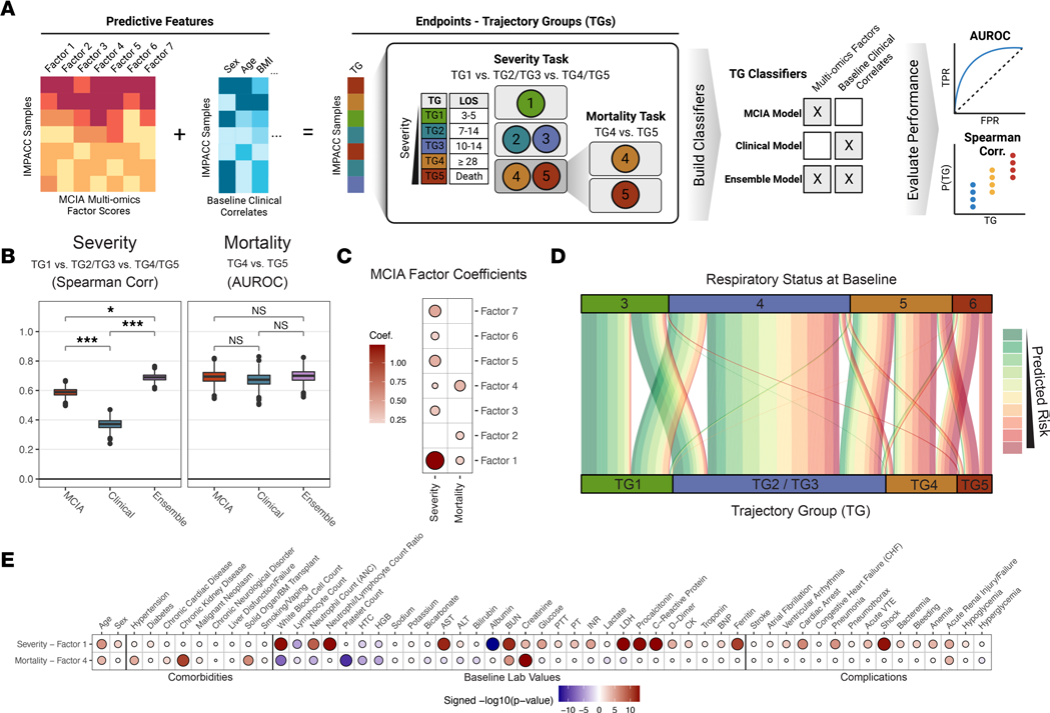
Multiomics factor prediction results. (**A**) Prediction schema (created with BioRender.com). (**B**) Box plots of Spearman correlations and AUROC values for the severity task [TG1, TG2/TG3, TG4/TG5 vs. Probability(TG4/5)] and mortality task [TG4, TG5 vs. Probability(TG5|TG4/TG5)] across bootstrapped iterations for each baseline model for the testing cohort. Significance was calculated by standard normal approximation of bootstrapped differences between models. (*P ≤ 0.05 and ***P ≤ 0.0001). (**C**) Dot plot of coefficients for the MCIA prediction model, with dot size and color representing magnitude and direction, respectively. (**D**) Alluvial plot showing the distribution of testing cohort individuals in each TG linked to their initial baseline respiratory status. The line color represents the predicted risk from the MCIA model. (**E**) Dot plot of *P* values from linear mixed-effects models with enrollment site as a random effect. Sex and age were further adjusted as fixed effects when associating baseline factor scores with various baseline clinical measurements, complications during hospital stay, and comorbidities in the training cohort, with dot size and color representing significance and direction, respectively. Values are only shown for adj. *P* ≤ 0.05.

We focused on 2 clinically relevant objectives to stratify disease severity using baseline multiomics profiles (26–28) (Figure 2A): predicting disease severity (identifying factors separating TG1 versus TG2/TG3 versus TG4/TG5; referred to as the “severity task”) and predicting fatal illness among critically ill participants (identifying factors separating TG4 versus TG5; referred to as the “mortality task”). To address the high dimensionality of the data, we first constructed low-dimensional multiomics factors using multiple co-inertia analysis (MCIA) (29) of the 539 IMPACC participants in our training cohort. The dimension reduction combined 27,320 analytes (92 for SPT; 210 for PPT; 1,430 for PPG; 722 for PMG; 12,408 for PGX; 12,458 for NGX) into multiomics factors that contained covarying patterns across assays.

We developed prediction models using multiomics factors from baseline samples (MCIA model) for each task to identify multiomics factors at hospital admission that correlated with clinical TGs. We also trained models using 28 clinical characteristics (clinical model) and combined multiomics factors and clinical characteristics (ensemble model) to assess and compare the performance across models (see Supplemental Methods). To validate these trained models, we applied the fitted MCIA factor construction and prediction model to the test cohort of 613 independent IMPACC participants first reported here and evaluated the resulting classification accuracy (Figure 1, C–E).

Both the MCIA model and the ensemble model outperformed the clinical model on the severity task, as measured by Spearman correlation (MCIA: ρ = 0.59; ensemble: ρ = 0.69; clinical: ρ = 0.37), and statistical significance was determined through the bootstrap procedure (*P* < 1 × 10^–6^) (Figure 2B). Inspection of the MCIA model coefficients for the severity task revealed a remarkable contribution from multiomics factor 1 (Figure 2C and Supplemental Figure 1A). The MCIA and ensemble models showed performance on the mortality task comparable to that of the clinical model, as measured by the area under the receiver operating characteristic curve (AUROC) (MCIA: AUROC = 0.69; ensemble: AUROC = 0.70; clinical: AUROC = 0.67) (Figure 2B). While MCIA and the clinical models displayed similar prediction performances for the mortality task, the MCIA model could provide insights into the immune program that distinguished TG4 and TG5. Key multiomics factors for the mortality task included multiomics factor 4 as the most salient, followed closely by factors 1 and 2 (Figure 2C and Supplemental Figure 1A). The MCIA models also achieved higher prediction accuracy than did the clinical models for separating each TG group from the others using the aggregated predictions (Supplemental Figure 1C).

We further investigated whether the multiomics factors could significantly improve the prediction of COVID-19 disease progression while controlling for respiratory status upon hospital admission (3 = no supplemental oxygen, 6 = invasive mechanical ventilation and/or extracorporeal membrane oxygenation) (5). We derived a predicted risk from the MCIA model for each participant at the time of hospital admission (the geometric mean of the probability of being critically ill and experiencing fatal illness, see Supplemental Methods) and evaluated its significance for predicting TGs while controlling for baseline respiratory status. Remarkably, the MCIA-derived risk score was associated with TGs (Figure 2D), even when evaluating only among those with moderately (baseline score in [3, 4], *P* = 6.9 × 10^–8^) and those with severely impaired baseline respiratory statuses (baseline score in [5, 6], *P* = 6.6 × 10^–11^), suggesting that the baseline molecular signatures captured by MCIA factors provide additional insight into a COVID-19 course trajectory not fully captured by the baseline respiratory status.

### Multiomics factors exhibit diverse associations with clinical profiles.

Factor 1 and factor 4 were identified as the strongest contributors to the severity and mortality tasks, respectively (Figure 2C). We refer to factor 1 as the “severity factor” and factor 4 as the “mortality factor” hereafter. Each of these factors represents a coordinated immune program involving correlated metabolite, protein, and gene profiles. We identified diverse significant associations (multiple testing adjusted [adj.] *P* < 0.05) between these 2 factors and comorbidities, clinical laboratory testing, and complications during the hospital stay for the initial 539 participants (Figure 2E). The severity factor showed strong positive associations with baseline total WBC (adj. *P* = 6.6 × 10^–14^) and neutrophil (adj. *P* = 1.3 × 10^–9^) counts, while being negatively associated with the lymphocyte count (adj. *P* = 2.0 × 10^–5^). We also noted significant associations with baseline values of several acute-phase reactants (adj. *P* < 0.05), including C-reactive protein (CRP), lactate dehydrogenase (LDH), ferritin, procalcitonin, and albumin, and with D-dimer, prothrombin time (PT), partial thromboplastin time (PTT), and international normalized ratio (INR), which are used to measure blood clotting functions. Moreover, the severity factor was predictive of high-acuity complications such as shock (adj. *P* = 1.3 × 10^–13^) and cardiac arrest (adj. *P* = 2.4 × 10^–7^). In contrast, the mortality factor demonstrated a strong negative association with baseline platelet (adj. *P* = 2.4 × 10^–11^) and total WBC (adj. *P* = 2.0 × 10^–8^), neutrophil (adj. *P* = 9.6 × 10^–5^), and lymphocyte (adj. *P* = 1.7 × 10^–2^) counts and strong positive associations with baseline serum creatinine and preexisting chronic kidney disease, hypertension, and solid organ transplantation (adj. *P* < 0.05). Regarding its association with complications during the hospital course, the mortality factor was most positively associated with acute renal failure (adj. *P* = 8.1 × 10^–5^).

### The severity factor unravels broad immune dysregulation as hallmarks of severity.

**Figure 3:**
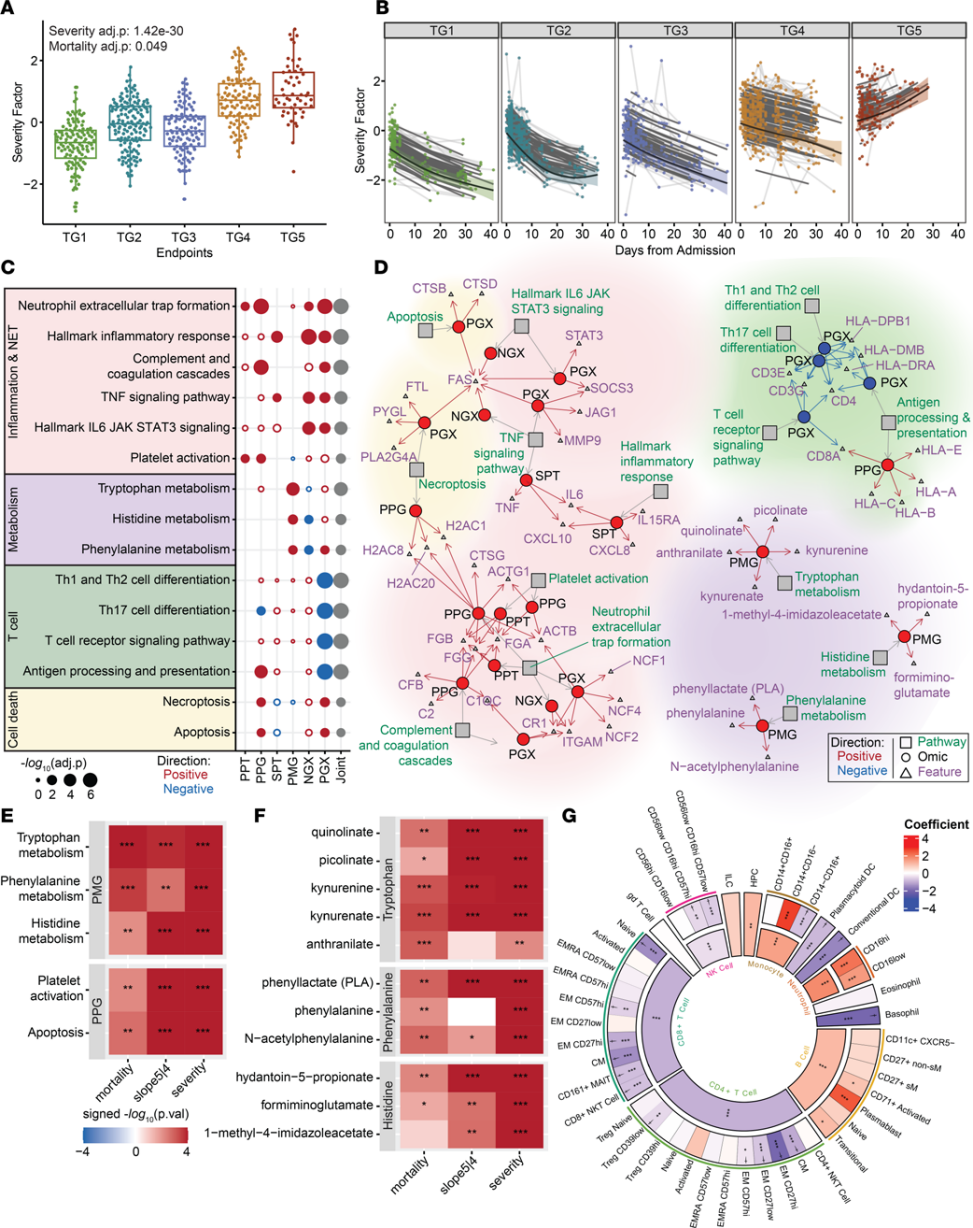
The severity factor increased in severe COVID-19. (**A**) The severity factor scores across clinical TGs at baseline (severity adj. *P* = 1.4 × 10^–30^, mortality adj. *P* = 0.049). (**B**) Longitudinal trajectory of the severity factor for different clinical TGs (mortality slope adj. *P* = 7.1 × 10^–15^). The shaded region denotes a 95% CI from a generalized additive mixed model of the fitted trajectory (thick black line), thin black lines show individual participant-fitted models, and light gray lines connect the participants’ time points. (**C**) Pathway enrichment of the severity factor. (**D**) Network of enriched pathways and selected high-contribution features. The full list of associated features is in Supplemental Table 5. (**E**) Heatmaps of differential expression tests for pathways in **C** that showed baseline separation between TG4 and TG5 with linear mixed-effects modeling. (**F**) Heatmap of differential expression tests of leading-edge metabolites from metabolism pathways in **E**. (**G**) Regression coefficients from linear mixed-effects modeling between the severity factor and normalized cell frequencies from whole blood (CyTOF) of both parent and child populations. Daggers indicate a significant association between the reduction of a child cell population frequency, which is significantly associated with the severity factor and severity factor apoptosis signaling in PGX (mortality/severity = baseline mortality/severity task, slope5|4 = TG5 vs. TG4 longitudinally; *P ≤ 0.05,**P ≤ 0.01,***P ≤ 0.001; joint = aggregated *P* value across omics).

While the severity factor and mortality factor were identified by the 2 prediction tasks using baseline omics measurements, we constructed the multiomics factors using samples from all visits to capture multiomics covariations at the baseline visit and over time. At the baseline visit, the severity factor displayed a significant increase with severity and mortality among the initial 539 critically ill participants (Figure 3A, severity adj. *P* = 1.4 × 10^–30^, mortality adj. *P* = 0.049, Supplemental Table 4), with the difference between groups increasing over time (Figure 3B). The more moderate groups (TG1–TG3) showed a sharp reduction in the severity factor levels over time compared with the more severe groups (TG4 and TG5) (adj. *P* = 5.2 × 10^–28^). Interestingly, while patients in the critical group who survived (TG4) also exhibited a gradual decrease in the severity factor over time, those in the critical group who died from the disease (TG5) displayed a significant increase in the severity factor level (Figure 3B, mortality slope adj. *P* = 7.1 × 10^–15^). These observations suggest that the severity factor captured features that were not only linked with overall COVID-19 severity but also associated with death among critically ill participants after hospitalization.

We defined high-contribution features for a factor as those highly correlated with this factor (see Methods) and used them to characterize the associated immune program. The severity factor showed strong covarying patterns across the different assays with 58 secreted cytokines/chemokines (of 92), 124 targeted plasma proteins (of 210), 685 global plasma proteins (of 1,430), 366 plasma metabolites (of 722), 3,637 PBMC genes (of 12,408), and 768 nasal genes (of 12,458) identified as high-contribution features (Supplemental Table 3). To characterize the biological dynamics underlying the severity factor, we used a minimum hypergeometric test (mHG) to assess the enrichment of known biological pathways among the high-contribution features of the factor, with enrichment being either positive or negative, corresponding to pathways directly and inversely associated with the factor. The pathway databases are composed of many broad and redundant biological pathways with highly overlapping gene sets (30). To handle this redundancy, biological pathways significantly enriched (adj. *P* < 0.1) in the severity factor were clustered on the basis of shared leading-edge features and separated into 4 major functional categories: inflammation, T cell activity, cell death, and dysregulated metabolism of essential amino acids (Supplemental Figure 2A and Supplemental Table 5). The joint multiomics enrichment was calculated by aggregating mHG *P* values from different omics within each functional category for prioritization (Supplemental Figure 2B; see also Supplemental Methods). The representative pathways from each of the 4 functional categories that reflected specialized biological functions with the most significant aggregated *P* values were selected for further evaluation (Figure 3C). The highlighted functions have been associated with COVID-19 disease severity previously in other study cohorts, but understanding of their coordination remains a challenge.

### Dysregulation of essential amino metabolism as an early hallmark of mortality among critically ill patients.

The severity factor was characterized by increased plasma metabolites from tryptophan catabolism (kynurenine and its derivatives anthranilate and kynurenate; adj. *P* = 1.10 × 10^–4^), phenylalanine metabolism (phenylalanine and its derivatives phenyllactate and *N*-acetylphenylalanine; adj. *P* = 0.056), and histidine metabolism (precursors of glutamate hydantoin-5-propionate, formiminoglutamate, and 1-methyl-4-imidazoleacetate; adj. *P* = 0.0247) (Figure 3, C and D). Notably, the observed metabolic dysregulations not only were associated with overall disease severity at baseline (Figure 3E, severity *P* < 0.05, Supplemental Table 6) but also demonstrated the most power in separating TG4 and TG5 at baseline among the pathways in Figure 3C (Figure 3E, mortality *P* < 0.05, Supplemental Figure 3A, and Supplemental Table 6). This early elevation was particularly interesting because participants in TG4 and TG5 had similar disease severities at the time of hospital admission (Figure 1B). Furthermore, the metabolic dysregulations were persistently elevated in TG5 participants throughout hospitalization (Figure 3E, slope 5|4 *P* < 0.05, and Supplemental Table 6) and were associated with the diverging severity factor kinetics between TG4 and TG5 (Supplemental Figure 2C). These suggest that dysregulations of essential amino acid metabolisms, including the overaccumulation of downstream products of essential amino acids such as kynurenine and phenylalanine substrates (Figure 3F), may play a key role in COVID-19 severity and contribute to the sustainment of the broad immune dysregulation captured by the severity factor in fatal illnesses at later stages.

To further evaluate the potential contribution of metabolic dysregulation in plasma to the systemic immune dysregulation, we conducted an interomics association analysis for direct association of the plasma metabolites with dysregulated cytokines and biological pathways (enriched pathway activities) from proteomics and transcriptomics assays across blood and nasal compartments (see Methods). Indeed, tryptophan, phenylalanine, and histidine pathways showed strong associations with high-contribution soluble proteins, with CD274, CD40, CX3CL1 (fractalkine), and IL15RA exhibiting the strongest average associations, even after controlling for demographic covariates, TGs, and visits (adj. *P* < 0.01). (Supplemental Figure 4A). *IDO1*-driven tryptophan breakdown correlates with the release of *HGF* (31) and heightened *IL10* (32), *IL6* (33), and *TNF* levels (34), all of which were significantly associated (Supplemental Figure 4A). Additionally, the metabolic pathways exhibited strong negative associations with T cell and antigen presentation pathways in PBMC transcriptomics (Supplemental Figure 4A). Tryptophan deprivation has been shown to sensitize T cells to apoptosis and inhibit T cell proliferation (35–37), while the tryptophan catabolite kynurenine induces Treg development and suppresses cytotoxic T cell responses (38, 39). Similarly, phenylalanine metabolism has been implicated in regulating the suppression of T cell immune responses (40).

### T cell lymphopenia is associated with increasing COVID-19 severity.

The severity factor was characterized by decreased signatures of T cell activities in PBMC transcriptomics (e.g., the T cell receptor signaling pathway [adj. *P* = 7.6 × 10^–7^] and the antigen processing and presentation pathway [adj. *P* = 9.2 × 10^–7^], including genes coding for the T cell receptor complex, such as *CD4*, *CD3E*, *CD3G*, and *CD8A,* genes involved in TCR signal transduction such as *NFATC13* and *LCK*, and genes coding for the MHC class II molecules *HLADPB1*, *HLADRA*, and *HLADMB*) (Figure 3, C and D). Reduced T cell–associated gene expression likely reflected the persistent T cell lymphopenia observed in TG5 participants (Supplemental Figure 2C). To validate this finding, we used linear mixed-effects modeling to evaluate for relationships of whole blood CyTOF cell frequencies with the severity factor and confirmed that the factor was associated with lower circulating T cells (Figure 3G). These results were also corroborated by the clinical association of lower absolute lymphocyte counts with the factor (Figure 2G) and were consistent with a targeted analysis of transcriptomics signatures of T cells from the literature (41–43) (Supplemental Figure 3B). Additionally, PBMC transcriptomics signatures of Th1, Th2, and Th17 cell differentiation pathways exhibited a downward trend over time in TG5 after adjusting for T cell frequencies (Supplemental Figure 3C), potentially reflecting dysregulated T cell functions besides lymphopenia.

Interestingly, the severity factor was also positively enriched in cell death pathways including necroptosis (including the genes *PLA2G4A*, *PYGL*, and *FTL*, coding for inducers of ROS in PBMC transcriptomics; adj. *P* = 0.052) and apoptosis (including the cathepsin genes *CTSD* and *CTSB*, and the gene coding for the cell death receptor *FAS* in PBMC transcriptomics; adj. *P* = 0.087). Apoptotic pathway activity was positively associated with increased levels of several high-contribution soluble proteins involved in regulating cell death (Supplemental Figure 3D) such as *CASP8* (44, 45), *CD274* (*PDL1*) (46), and *TNF* (47) and was negatively associated with the CD4^+^/CD8^+^ T cell frequencies in whole blood measured by CyTOF (Figure 3G, daggers). Our results suggest that increased induction of apoptosis, which could be modulated by inflammatory cytokines (Figure 3D), might contribute to the loss of circulating T cells observed in patients with the most severe disease (5).

These findings are also consistent with the negative associations between T cell pathways and elevated tryptophan metabolism, which could reflect T cell apoptosis and dysregulated T cell signaling. Interestingly, increased protein levels of *CD274* (*PDL1*), a ligand for the inhibitory receptor programmed cell death 1 (PD-1) on T cells (46), was the top associated feature with the observed metabolic pathways enriched in the severity factor (Supplemental Figure 4A). The interomics association among essential metabolite dysregulation, *CD274*, and T cell pathway activity revealed an orchestrated suppression of T cell responses across the 3 assays (metabolomics, serum proteomics, and transcriptomics).

### An inflammation and NET formation network associates with worse outcomes.

The severity factor was positively enriched in diverse inflammatory pathways across proteomics and transcriptomics of both the nasal and blood compartments. Within these, inflammatory markers previously associated with COVID-19 severity were among the high-contribution features of the severity factor (Figure 3D and Supplemental Table 3), including *IL6*, *CXCL10*, and *CXCL8* (*IL8*) proteins in serum. Proinflammatory soluble proteins are known to modulate metabolism (48, 49), transcription (50, 51), and other cellular activities (52–54). Similarly, our analyses revealed strong associations of proinflammatory soluble proteins with transcriptomic, proteomic, and metabolomic pathways and cellular compositions in blood (Supplemental Figure 4, A and B).

Markers of NET formation were strongly enriched in the severity factor across multiple omics (including the protease cathepsin G [*CTSG*]; the histone *H2AC20*, *H2AC1*, and *H2AC8* proteins in plasma; and genes encoding the neutrophil NADPH oxidase factors *NCF1*, *NCF2,* and *NCF4* in PBMC transcriptomics; adj. *P* = 7.6 × 10^–7^). In support of an increase in NET formation with increasing disease severity, the severity factor showed positive enrichment of upstream inducers of NET formation, such as IL-6 signaling (cytokine IL-6 and the genes *SOCS3* and *STAT3* in the PBMC transcriptomics; adj. *P* = 1.4 × 10^–4^), platelet activation (fibrinogens FGA, FGB, and FGG in plasma proteins [ref. 55]; adj. *P* = 0.02), and complement and coagulation (including the plasma proteins C2, CFb, and C1QC and genes coding for the complement receptor 1 [*CR1*] and *ITGAM* in PBMC transcriptomics; adj. *P* = 2.5 × 10^–6^). Notably, the platelet activation pathway showed significant elevation in TG5 compared with TG4 at baseline (Figure 3E and Supplemental Figure 3A), supporting the possibility that platelet activation might trigger distinct kinetics of NETosis in TG4 versus TG5. Also enriched were intracellular pathways triggered during NET formation, such as actin degradation (actins *ACTB* and *ATCG1* in the plasma proteins [ref. 56]) and TNF/NF-κB signaling (cytokine *TNF* and NF-κB target genes *MMP9*, *FAS*, *JAG1*; adj. *P* = 1.6 × 10^–4^), as were receptors expressed by and cytokines produced by neutrophils, including receptor *IL15RA* (57) and cytokines *CXCL8* (*IL8*) and *IL17A* (58, 59) (Figure 3, C and D). Notably, *CXCL8* is a neutrophil chemoattractant, indicating a potential increase in neutrophil production or greater recruitment in patients with more severe disease. These enriched inflammatory pathways were elevated over time uniquely in the mortality group, suggesting prolonged and unresolved inflammation associated with neutrophils preceding death (Supplemental Figure 2C).

**Figure 4:**
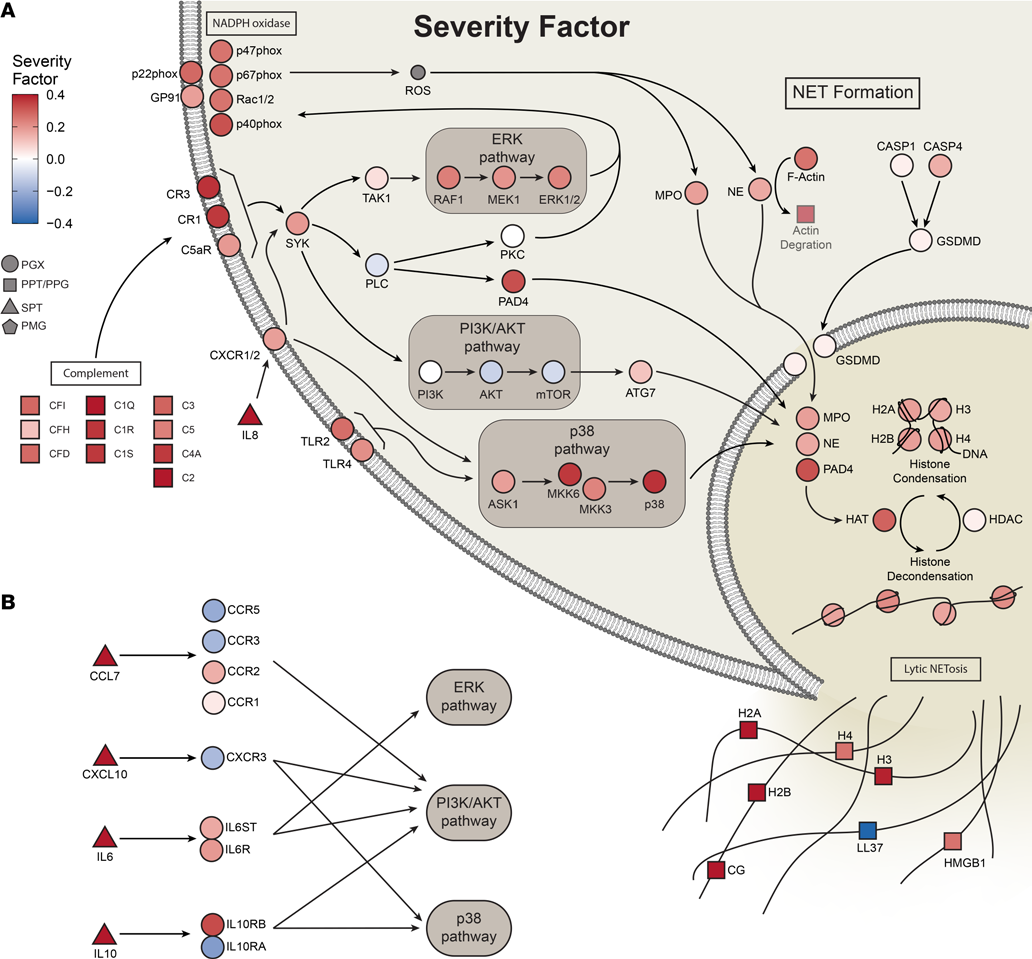
Integrative multiomics network identifies upstream regulators and mediators of NET formation. (**A**) Broad elevation of transcriptomics and proteomics features in NET formation and complement in the severity factor. Pathway connections are from the Kyoto Encyclopedia of Genes and Genomes (KEGG) NET formation pathway. (**B**) Top cytokines in the severity factor, when bound to their receptors, trigger downstream signaling pathways, including ERK and p38 signaling pathways, and are important in NET formation.

The enrichment of NET formation signatures also included ERK and p38 signaling pathways in transcriptomics, forming core inflammatory signaling pathways triggered by many high-contribution cytokines grouped as “cytokines produced by macrophages” and “cytokines produced by neutrophils” (6) (Figure 4A). Notably, among these cytokines, *IL10*, *IL6*, *CXCL10*, and *CXCL7* were the most strongly associated with the severity factor and are known to elicit ERK and p38 signaling (Figure 4B). Along with enrichment of ERK and p38 signaling, we also noted significant enrichment of activator protein 1–regulated (AP-1–regulated) genes in the PBMC transcriptomics, and the transcriptional factor AP-1 is a downstream target of ERK and cytokine signaling (Supplemental Figure 3E). AP-1 has been previously highlighted as a top feature of COVID-19–associated severity along with p38 and MAPK signaling (7). Receptors for the top 4 high-contribution cytokines in the severity factor were detected in PBMC transcriptomics (Figure 4B), with *IL6* receptor components positively enriched in the factor. However, other receptors such as *CXCR3*, the receptor for *CXCL10*, were weakly positively or negatively associated with the severity factor, reflecting a potential reduction of certain cell types in the circulating PBMCs. Notably, reduced *CXCR3* expression was consistent with lymphopenia, as suggested by reduced clinical absolute lymphocyte counts (Figure 2G). Such mixed correlations of receptors were also observed for other cytokines with high contribution to the severity factor (Supplemental Figure 4C).

To complement our pathway analysis, we investigated the association of the severity factor with immune cell frequencies in whole blood measured by CyTOF. We evaluated the overlap of severity factor high-contribution genes with transcriptomic markers of immune cells from blood and nasal tissue (41–43). Consistent with the enrichment of NET formation, the severity factor positively correlated with neutrophil frequencies in whole blood and was enriched for transcriptomic markers of neutrophils in the nasal transcriptomics (Figure 3G and Supplemental Figure 3A). The severity factor was also positively correlated with monocytes and significantly enriched in transcriptomic markers of monocytes (Supplemental Figure 3B), suggesting that monocytes may play a critical role in the inflammatory response identified and possibly promote NETosis.

### The mortality factor reveals B cell and plasma Ig reduction as early hallmarks of mortality among critical illness.

**Figure 5:**
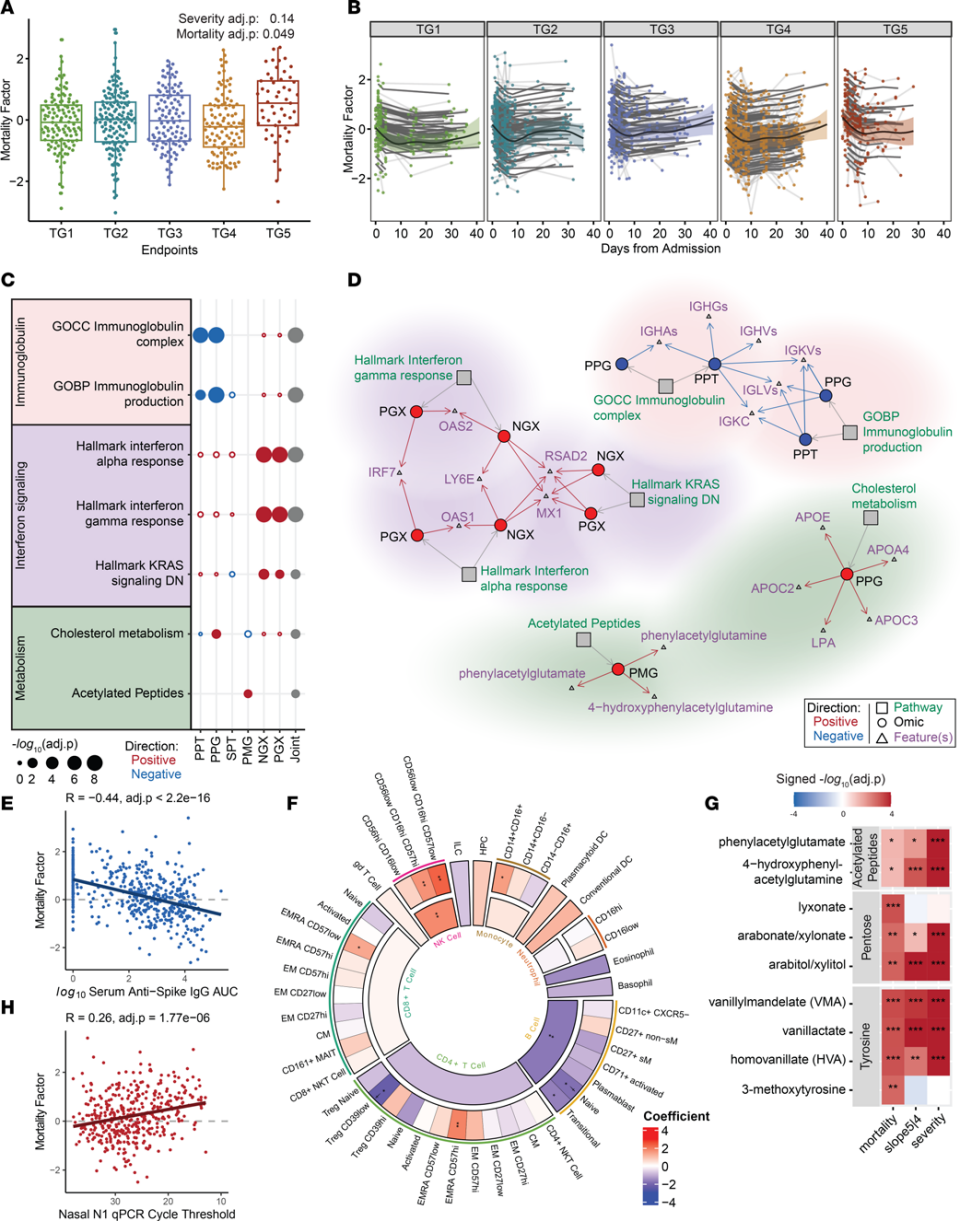
Multiomics mortality factor enriched for antibodies, IFN signaling, and cellular metabolic changes. (**A**) Mortality factor scores across clinical TGs at baseline (severity adj. *P* = 0.14, mortality adj. *P* = 0.049). (**B**) Longitudinal trajectory of the mortality factor for different clinical TGs. The shaded region denotes a 95% CI of the fitted trajectory (thick black line), thin black lines show individual participant-fitted models, and light gray lines connect the participants’ time points. (**C**) Functional pathway enrichment of the mortality factor revealed downregulation of Igs, upregulation of the IFN response, cholesterol metabolism, and acetylated peptides. (**D**) Network of enriched pathways in **C** and top selected high-contribution features. The full list of associated features is given in Supplemental Table 7. (**E**) Spearman correlation test between the mortality factor and serum anti–spike IgG antibody using baseline samples; *P* values were calculated from a linear mixed-effects model controlling for TG, sex, and age. (**F**) Regression coefficients from linear mixed-effects modeling of the mortality factor with normalized cell frequencies from whole blood (CyTOF) of both parent and child populations. Daggers indicate a significant association between the reduction of a child cell population frequency, which is significantly associated with the mortality factor and severity factor apoptosis signaling in PGX. (**G**) Differential expression tests via mixed-effects modeling of leading-edge metabolites in highlighted metabolomic pathways. (**H**) Spearman correlation coefficient between the mortality factor and nasal SARS-CoV-2 quantitative PCR (qPCR) Ct using baseline samples; *P* values were calculated from a linear mixed-effects model controlling for TG, sex, and age (mortality/severity = baseline mortality/severity task, slope5|4 = TG5 vs. TG4 longitudinally; *P ≤ 0.05, **P ≤ 0.01, ***P ≤ 0.001; joint = aggregated *P* value across omics).

The mortality factor was significantly higher at baseline for those who died within the first 28 days of hospitalization (TG5) compared with critically ill participants who survived (TG4) (Figure 5A, severity adj. *P* = 0.14, mortality adj. *P* = 0.049, Supplemental Table 4). Over time, the relative levels of the mortality factor dropped and were sustained at low levels in all groups (Figure 5B). Hence, this factor represents a mortality-related immune state at hospitalization and stratifies mortality for other critically ill patients.

The features with the highest contribution to the mortality factor were primarily plasma proteins and metabolites (Supplemental Table 3), including 89 targeted plasma proteins, 289 global plasma proteins, and 172 plasma metabolites. Only 14 secreted cytokines/chemokines (of 92), 42 PBMC genes (of 12,408), and 31 nasal genes (of 12,458) were highly contributing features to this factor (Supplemental Table 3). At baseline, there were 7 enriched pathways (adj. *P* < 0.1, Supplemental Table 7) that separated TG5 and TG4 in least 1 assay (*P* < 0.05, Figure 5C, Supplemental Figure 5A, and Supplemental Table 8).

The most prominent characteristic of the mortality factor was a reduction in Igs in the proteomics assays, including heavy- and light-chain variable regions and constant regions from multiple isotypes (e.g., IGHGs, IGHAs, IGHVs, IGKVs, IGLVs) (Figure 5D, adj. *P* = 2 × 10^–8^ for the Ig complex in PPT; 1.48 × 10^–9^ for the Ig complex in PPG; 0.00637 for Ig production in PPT; 3.8 × 10^–9^ for Ig production in PPG). The mortality factor was also negatively associated with serum anti–spike IgG (Figure 5E, adj. *P* < 2.2 × 10^–16^). Furthermore, this factor was negatively correlated with the frequency of total circulating B cells, particularly naive and transitional B cell subsets measured by CyTOF (Figure 5F, adj. *P* = 4.59 × 10^–19^), and total circulating B cells were also positively correlated with plasma Ig (Supplemental Figure 5B, adj. *P* = 2.34 × 10^–8^). The 42 high-contribution PBMC genes were also negatively enriched for transcriptomic markers of B cells (Supplemental Figure 5C, adj. *P* = 3.00 × 10^–10^). These findings suggest that the mortality factor captures a lower level of B cell activity, plasma Igs, and anti–spike IgG at the baseline in patients who died within the first 28 days following hospitalization (Supplemental Figure 5, D and E). The decline in B cells could partially reflect increased apoptosis, especially of naive B cells, as suggested by the positive association with the apoptosis pathway constructed from the severity factor (Figure 5F, adj. *P* ≤ 0.05).

### Dysregulated IFN responsiveness and cellular metabolic changes indicate mortality.

Alongside the reduced Ig and B cell activity, the mortality factor exhibited a strong positive enrichment of IFN-stimulated genes (ISGs) in PBMC transcriptomics (*OAS1* and *OAS2*, encoding viral RNA sensors, and *IRF7*, Figure 5D) and in nasal transcriptomics (*MX1*, *RSAD2*, *LY6E*, encoding antiviral immune genes). Further examination revealed that the leading-edge ISGs are enriched in antiviral rather than proviral functions (60, 61). IRF-regulated and STAT-transcribed genes were also positively enriched in the mortality factor across both nasal and PBMC transcriptomics (Supplemental Figure 6A), confirming the propagation of IFN signal through transcriptional factor activity. Along with elevated JAK/STAT IFN signaling, known inhibitors of IFN signaling (IFN inhibitors) (62), including *USP18*, *SOCS1*, and *PIAS4*, were positively enriched in the mortality factor (*P* = 0.030), suggesting the possibility of dysregulated IFN responsiveness in critically ill patients.

The mortality factor was also enriched in acetylated peptides (4-hydroxyphenylacetylglutamine, phenylacetylglutamate, and phenylacetylglutamine) in the plasma metabolites (adj. *P* = 0.08) and cholesterol synthesis–related plasma proteins (adj. *P* = 1.75 × 10^–4^), including *APOC2*, *APOC3*, *APOA4*, *APOE*, and *LPA* (Figure 5, C and D). To comprehensively explore soluble markers contributing to the early separation between TG4 and TG5 in the mortality factor, we performed enrichment analysis of high-contribution metabolites and cytokines that also separated TG4 and TG5 at baseline (*P* < 0.05, Supplemental Table 8). We identified positive enrichment of pentose metabolism (including lyxonate, arabitol/xylitol, arabonate/xylonate, adj. *P* = 0.07) and tyrosine metabolism (including VMA, HVA, vanillactate, 3-methoxytyrosine, adj. *P* = 0.07), in addition to acetylated peptides, whose leading-edge metabolites showed separation between TG4 and TG5 at hospital admission (Figure 5G and Supplemental Table 8). An interomics association analysis further revealed significant associations between the 3 highlighted metabolomic pathways and proteomics functions, including Ig complex reduction and cholesterol metabolism elevation, as well as soluble proteins *CST5*, *CX3CL1*, *CCL25*, *CSF1*, and *KITLG* (adj. *P* < 0.01, Supplemental Figure 6B).

We note that the mortality factor was also negatively correlated with the platelet count on hospital admission (Figure 2F, adj. *P* = 2.36 × 10^–11^), which was consistent with the observed positive enrichment in complement and coagulation pathways in plasma proteomics (including *FGA*, *FGB*, *C3*, *C4A*, *C4B*, and *C9*, adj. *P* = 0.007 [PPT], adj. *P* = 0.03 [PPG], Supplemental Table 7). Additionally, the mortality factor was positively enriched in participants with preexisting chronic kidney disease and renal complications during the hospital stay. Moreover, clinical laboratory testing demonstrated a positive correlation between the mortality factor and baseline clinical creatinine, a biomarker of kidney function, in addition to creatine being a high-contribution feature to the mortality factor in plasma metabolomics (Supplemental Table 3). Creatinine was noted to have a strong positive correlation with acetylated peptides, cholesterol metabolism, tyrosine metabolism, and pentose metabolism (ρ = 0.46, 0.24, 0.53, 0.53; adj. *P* = 2.10 × 10^–65^, 6.69 × 10^–18^, 2.98 × 10^–92^, and 9.82 × 10^–91^, respectively, Supplemental Figure 6C).

### The mortality factor reveals a dysregulated viral response immune cascade.

**Figure 6:**
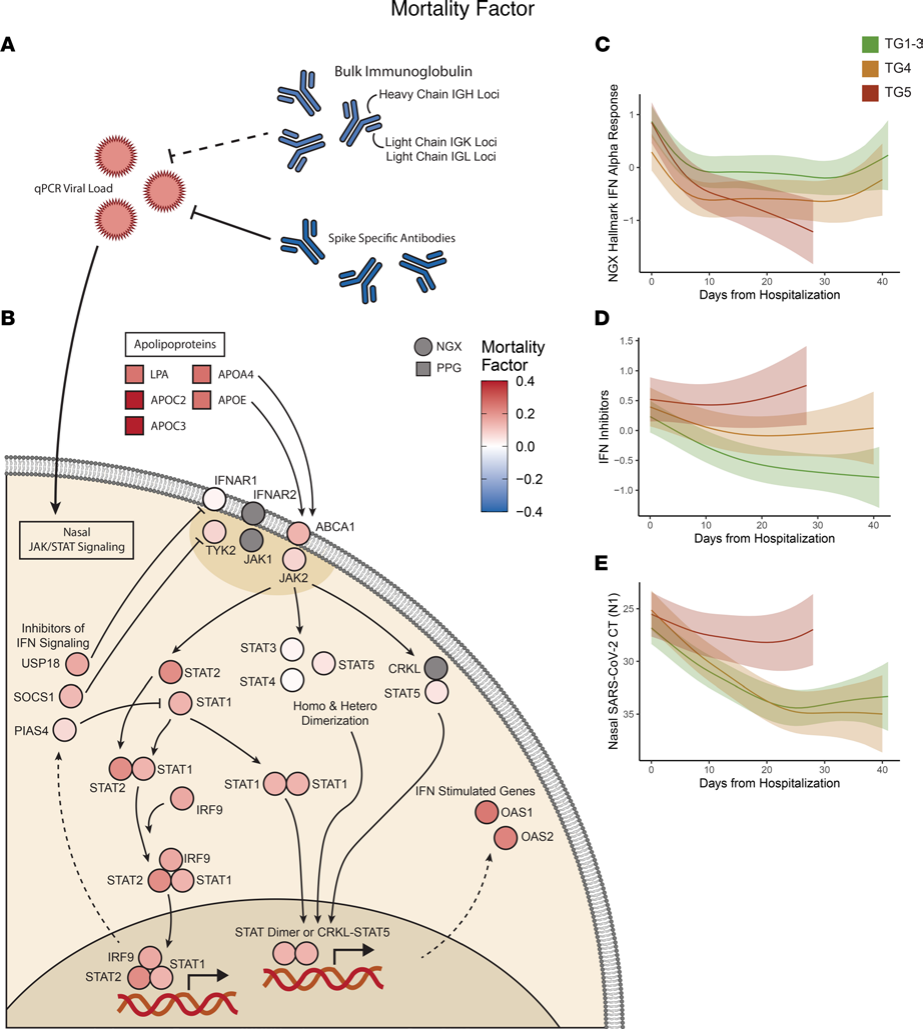
Virus-centered integrative multiomics network of the mortality factor. (**A**) Positive association of nasal SARS-CoV-2 viral load and inverse associations of total and SARS-CoV-2–specific antibodies were top features of the mortality factor. (**B**) JAK/STAT IFN signaling was positively associated with the mortality factor and viral load, and IFN signaling inhibitors were also positively associated with the mortality factor, potentially contributing to the dysregulation of IFN responsiveness and uncontrolled viral load in TG5 despite early ISG elevation. The elevation of apolipoproteins from the plasma proteomics may also have contributed to the heightened STAT activity. Longitudinal trajectories from generalized additive mixed-effects modeling of (**C**) hallmark IFN-α response genes in the NGX, (**D**) IFN inhibitors in the NGX, and (**E**) nasal SARS-CoV-2 viral load determined from RT-qPCR Ct.

A reduction of total plasma Igs and elevation of IFN-stimulated genes were the most prominent features of the mortality factor, together with strong negative associations with serum SARS-CoV-2 antibody titers and a robust positive association with nasal viral loads (Figure 5H). This suggests that a dysregulated host immune cascade may have contributed to the failure of viral clearance in TG5. Notably, the total Ig level was strongly associated with the serum SARS-CoV-2 antibody titer levels and had an inverse relationship with viral load (Figure 6A and Supplemental Figure 6C, adj. *P* ≤ 0.05).

In contrast, both IFN pathway activity and IFN inhibitor levels showed a strong inverse relationship with serum SARS-CoV-2 antibody titers and total Igs and a positive correlation with viral loads in both nasal (Figure 6B and Supplemental Figure 6C) and PBMC (Supplemental Figure 7 and Supplemental Figure 6C) ISGs. The observed elevation of nasal ISGs in TG5 at baseline potentially reflected virus-associated IFN production. After adjusting for viral load, nasal ISG levels became more comparable between TG4 and TG5 (Supplemental Figure 6D, *P* = 6 × 10^–3^ before adjustment, *P* = 0.11 after adjustment, see Supplemental Methods). This adjustment accentuated the overall lower ISG expression levels in the TGs with more severe disease versus those with moderate disease (TG4/TG5 vs. TG1–3, Supplemental Figure 6D), consistent with the reduced IFN responsiveness among patients with severe disease observed in previous studies (13). Interestingly, type I IFN gene expression in nasal transcriptomics declined more rapidly in TG5 than in TG4, both before and after adjustment (Figure 6C and Supplemental Figure 6E, *P* = 9 × 10^–3^ before adjustment and *P* = 7 × 10^–3^ after adjustment). This substantially faster ISG decline in TG5 was accompanied by elevated expression of known inhibitors of IFN signaling (Figure 6D, adj. *P* = 0.0015), which was uniquely observed in TG5 and matched the viral load trend in TG5 (Figure 6E). These observations supported the possibility of dysregulated IFN responsiveness among critically ill participants, which was unresolved in those who succumbed to infection, suggesting that higher viral loads could trigger elevated IFN signaling that may counterproductively turn on a negative feedback loop to suppress IFN signaling before the virus is cleared (62, 63) and contributing to mortality (Figure 6, B–E). Additionally, plasma proteomics demonstrated elevation of apolipoproteins that can bind receptors capable of activating JAK signaling, possibly contributing to the heightened STAT activity (64) (Figure 6B).

Overall, our results suggest that mortality among critically ill patients could significantly associate with dysregulation of the host transcriptome, plasma metabolome, and proteome, as well as loss of circulating B cells, which was concomitant with a persistent viremia observed in the critically ill participants, who died as a result of the disease within the first 28 days.

## Discussion

Our study represents an extensive evaluation of patients hospitalized with COVID-19, encompassing a wide range of omics. As participants were studied before the widespread availability of SARS-CoV-2 vaccines, our study provides a unique perspective on the naive viral response. The use of a previously published study involving a training cohort of 539 participants and validation with a newly introduced 613-participant test cohort, which varied in demographics, clinical laboratory testing (Supplemental Table 2), and enrollment periods during the pandemic, enabled robust identification of multiomics factors associated with COVID-19 severity and mortality. These factors outperformed previously studied clinical features (5), single-omics analytes, and several single-assay COVID-19 molecular signatures reported in the literature (65–67) in predictive modeling (Supplemental Figure 1I).

**Figure 7:**
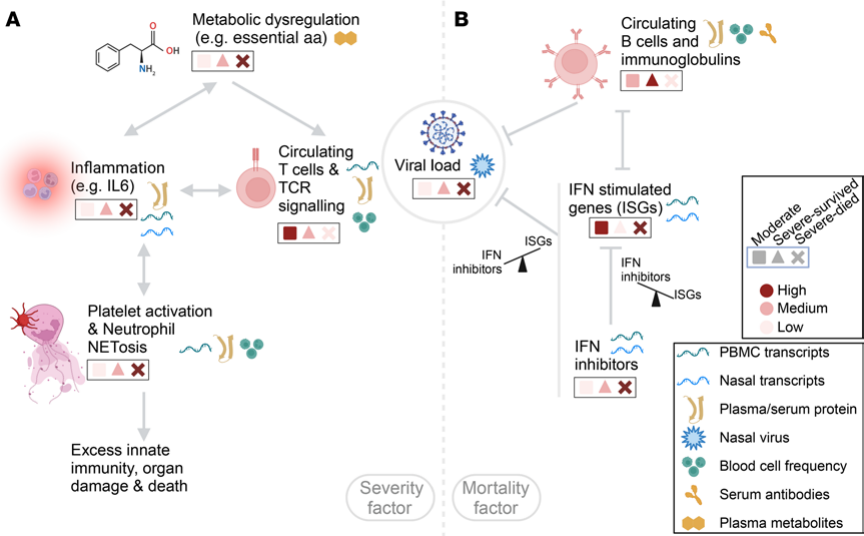
Summary of highlighted host immune programs. (**A**) The severity factor identified an integrated multiomics cascade associated with disease severity, characterized by dysregulated metabolisms, e.g., essential amino acid (aa) metabolism, elevated inflammatory soluble proteins and transcripts, an elevated signature of coagulation and NETosis, and reduced T cell circulation and signaling in patients with more severe disease. The dysregulated metabolisms potentially served as early modulators of this broadly dysregulated immune state. The links in this panel reflect hypotheses formulated on the basis of our findings. (**B**) The mortality factor revealed a virus-centered multiomics immune state as an early hallmark of mortality among the critically ill patients (ICU, ventilation, or mortality), including reduced Igs and B cell circulation, dysregulated IFN responsiveness, as suggested by elevated IFN inhibitor levels in both nasal and PBMC transcriptomics, along with persistently elevated viral loads in patients with fatal illness. This figure was created with BioRender.com.

A key severity factor (factor 1) captured the severity trend among all hospitalized participants (TG1 vs. TG2/TG3 vs. TG4/TG5) and distinguished participants in the critically ill group who survived during the first month (TG4) from those in the mortality group (TG5), with elevation over time uniquely in TG5 participants. In line with our prior work on clinical presentations of the TGs, the severity factor was significantly associated with older age, male sex, and comorbidities such as diabetes and hypertension. The factor illuminates a spectrum of immune changes across 6 omics platforms extending beyond mere cellular alterations and captures an increase in metabolites related to tryptophan, phenylalanine, and histidine pathways; enhanced signatures of inflammation coagulation and NET formation; heightened signatures of cell apoptosis; and a decrease in T cell signatures and circulating T cell numbers, which also showed strong associations with metabolic dysregulations and proinflammatory soluble proteins (Figure 7A). Elevated serum concentrations of multiple circulating cytokines and chemokines were associated with the severity factor, including elevation of 2 clusters of soluble proteins characterized by “cytokines produced by neutrophils” and “cytokines produced by macrophages,” as well as a negative association with the cluster “activators of cytotoxic NKs” (Supplemental Figure 4). Thus, the elevated longitudinal trend in TG5 could reflect an unresolved dysregulation and elevation of inflammatory cytokines in fatal illness when the cytotoxic antiviral activities of T cells and NK cells were insufficient to reduce viral burden. The negative association with activators of cytotoxic NKs cluster was consistent with previous analysis results from the targeted serum proteomics assay alone, which identified this cytotoxic NKs cluster as a marker of recovery (6).

A joint investigation of molecular signatures’ baseline levels and kinetics in the severity factor revealed that essential amino acid metabolism dysregulation potentially contributed to its distinct kinetics between TG4 and TG5. These amino acids, and their metabolites, act as important protein building blocks, key energy sources in metabolic pathways (e.g., Krebs cycle), and modulators of immunity (68). Our analysis also suggested the immune-modulatory role of metabolites associated with the severity factor, shown via strong associations of essential amino acid dysregulation and high-contribution cytokines produced by neutrophils and macrophages, as well as protein and transcriptomic pathways in the severity factor. For example, significant associations observed between *CD274* (programmed death ligand 1 [PD-L1]), T cell pathway activity, and dysregulated tryptophan metabolism suggested a coordinated suppression of T cell responses across metabolomics, plasma/serum proteomics, and transcriptomics. Essential amino acid metabolism dysregulation was significantly associated with *TNF*, *IL6*, *IL10*, *HGF*, and *CD40*, all of which were linked to IDO1-driven tryptophan breakdowns (31–34). Interestingly, metabolites resulting from IDO1-driven tryptophan breakdowns, such as kynurenine, kynureninate, and 3-hydroxykynurenine, were highly elevated in the severity factor, suggesting functional activity of *IDO1*. Additionally, *IDO1* participates in the phenylalanine catabolic pathway, resulting in the formation of phenylpyruvate. The functional activity of *IDO1* was also suggested by the positive correlation of phenylalanine pathway metabolites with *HPD*, *FAH*, and *SLC16A1* in PBMC transcriptomics, which are genes associated with the active conversion of phenylpyruvate from phenylalanine (69–71) (Supplemental Figure 3F). Furthermore, phenylalanine, tyrosine, and tryptophan metabolism has been previously reported to be dysregulated in patients with sepsis, suggesting this dysregulation may be elicited in response to multiple types of severe infections (72). These observations indicate that these metabolites could be evaluated clinically in early COVID-19 to assess disease severity, and intervention via modulation of nutrients may prove beneficial, with several clinical trials currently underway in patients with COVID-19 to evaluate amino acid supplementation (73–75).

Another highlighted factor, the mortality factor (factor 4), was substantially higher at baseline in participants in TG5 compared with those in TG4, and captured an immune program of dysregulated IFN signaling, an elevated nasal viral load, and a reduction in circulating B cells, bulk Ig, and SARS-CoV-2–specific serum IgG (Figure 7B). The mortality factor was significantly associated with older age and several comorbidities including chronic kidney disease, solid organ/bone marrow transplant, hypertension, and chronic cardiac disease but, notably, was not significantly associated with sex. The levels of both SARS-CoV-2–specific IgG and total plasma Ig were reduced in the mortality factor and were negatively associated with viral load. These findings suggest that B cell immune responses were important for controlling the virus among critically ill patients during early infection. In addition, the overall reduction in circulating Ig in participants who did not recover could contribute to general inflammation, as intravenous Ig (IVIG) therapy suppresses inflammatory pathology (76–78). Thus, the reduction of B cell activity and circulating Ig in patients who died from COVID-19 during the first month of hospitalization might not only have allowed higher viral replication in critical illness, which could have fueled additional inflammation, but could have also potentially promoted inflammation indirectly, resulting in tissue damage.

Antiviral ISG activity increased with the mortality factor and was positively associated with viral load, consistent with a virus-induced increase in type I IFNs (79). Remarkably, while ISG levels were relatively higher in the mortality group TG5 compared with TG4 at baseline, the expression of IFN-inhibitory genes in the upper respiratory tract was also elevated in TG4 and TG5 at baseline, which could suggest suppression of effective IFN signaling in critically ill patients. In addition to the elevated IFN-inhibitory genes in TG4 and TG5 at baseline, TG4 and TG5 had a higher proportion of participants with anti-IFN antibodies, which are associated with COVID-19 severity and can inhibit IFN and STAT signaling (17) (Supplemental Figure 6, F and G). When evaluated longitudinally, IFN-inhibitory genes were uniquely elevated in TG5, which could also contribute to the faster decline in ISGs in TG5 compared with other groups, leading to inadequate IFN signaling in the mortality group TG5. Notably, viral load remained high only in TG5 over time, suggesting failed control of viral clearance. The combined results from analyzing viral loads, Ig levels, and IFN signaling/inhibitor genes raise the possibility that, while IFN-induced viral clearance may be successful in patients with moderate disease (TG1–TG3), IFN responsiveness might be dysregulated, potentially due to elevated expression of IFN inhibitors driven by sustained high viral loads in the critically ill patients (TG4 and TG5). The observed dynamics of viral load, IFN, and antibodies supports the idea of early administration of antiviral and antibody therapies as an essential intervention to reduce mortality. The mortality factor stratified participants in TG4 versus TG5 primarily during the early stages of hospitalization, indicating the importance of timely intervention, as supported by clinical trial data on Paxlovid (80, 81). Several trials showed that early treatment with mAb therapies in outpatients reduced hospitalizations and severe disease when the mAb matched the virus variants in circulation (82–84). However, IVIG and convalescent plasma therapies failed to meet their primary endpoints in several acute COVID-19 clinical trials, with their roles remaining inconclusive (85–89). One potential explanation for the failure may be related to the timing of administration of these therapies and patient inclusion criteria in the trials, both of which could be critical factors for intervention, as suggested by our analyses.

The mortality factor was strongly associated with specific disease comorbidities including chronic kidney disease, immunosuppression, and hypertension, which relate to mortality factor high-contribution molecular markers such as creatinine. Additionally, acute renal disease, a frequent complication in critically ill patients, was linked to the mortality factor. This association may account for the observed increase in tyrosine and pentose metabolites, typically cleared by the kidneys, as well as the mortality factor’s correlation with elevated creatinine and blood urea nitrogen (BUN) levels (90–92). The tyrosine metabolites (HVA, VMA, and VLA) are also byproducts of catecholamine biosynthesis/degradation and are major terminal urinary metabolites converted from l-dopa, dopamine, and norepinephrine, which may reflect the use of exogenous pressors in ventilated patients (Supplemental Figure 3F). Furthermore, previous work has suggested that dopamine inhibits SARS-CoV-2 viral replication and stimulates type I IFNs, with SARS-CoV-2 possibly disrupting dopamine pathways which could also result in increased catecholamine downstream byproducts (93, 94).

Another interesting observation from comparing the severity and mortality factors is that “complement and coagulation” was a significantly enriched functional pathway in both factors despite their low correlation. Overlap between the factors could reflect different biological processes that influence the complement and the coagulation pathway activities. Although the 2 factors shared many leading-edge proteins, the severity factor was more strongly associated with classical complement-associated proteins (C1Q, C1R, C1S, C2), whereas the mortality factor had a more significant association with the alternative pathway (C3, FB, FD). The severity factor was enriched in inflammation, platelet activation, and NET formation, which are closely related to the complement and coagulation cascades (95). Using baseline clinical laboratory measures, we observed that the severity factor was positively associated with D-dimer, PTT and PT, suggesting a relationship with coagulopathy in patients with COVID-19 (96). In contrast, the mortality factor was negatively associated with platelet count and demonstrated no significant association with D-dimer, PT, or PTT. These associations collectively suggest that impaired coagulation is associated with severe disease, whereas prolonged thrombocytopenia may be predictive of mortality. Our findings contribute mechanistic evidence to the evolving concept of “immunothrombosis,” which refers to the complex interplay between immune cells, complement, coagulation factors, and NETs that are important in COVID-19–associated coagulopathy (12, 97, 98).

Our integrative longitudinal analysis formulates mechanistic hypotheses concerning temporal coordination, elucidating the heterogeneity in disease progression among hospitalized patients. Our expansive analysis across training and validation cohorts corroborates these hypotheses. Although this study does not facilitate further validation through functional assays, it establishes a substantive foundation for future in-depth investigations of detailed mechanisms behind disease heterogeneity.

### Limitations.

All participants in the IMPACC cohort were hospitalized for COVID-19 as part of the study design (23); therefore, our immune programs (MCIA factors) were not constructed using profiles from individuals with mild COVID-19 or healthy individuals and may be biased toward those with more severe COVID-19. However, many of our highlighted functions, such as NET formation, T cell lymphopenia, and tryptophan catabolism, have been identified in other cohorts with a full spectrum of COVID-19 severity (7, 28, 99–101). Similarly, vaccination and varying SARS-CoV-2 strains, both of which were not evaluated in the present study, may further alter the observed severity and mortality signatures. Furthermore, as nasal transcriptomics may not be reflective of the lower respiratory tract, additional work exploring omics directly from the lower respiratory tract may help elucidate how infection was contributing to the systemic changes observed in this study. In addition, the present study did not explore other questions of interest, including differences in immune responses by age, sex, COVID-19–associated comorbidities, medication, and post-acute sequelae of COVID-19, which were planned for future follow-up work. Still, the IMPACC deep multiomics immunophenotyping data present a uniquely rich resource for further investigations, including those not covered by this study. For example, while the severity factor showed many significant pathways in the enrichment analysis, we focused on the specialized biological functions that were corroborated by multiple assays except for metabolism functions due to the limitation of existing knowledge bases. Broader or single data set–restricted pathways, unexplored here, could illuminate additional complex aspects of COVID-19.

## Methods

### Sex as a biological variable.

Our study examined male and female participants. The cohort included 704 (61%) men and 448 (39%) women. We found that male sex was significantly associated with the severity factor, which was in line with men comprising a larger proportion of the TGs with more severe disease. To control for this imbalance, sex, in addition to age, were used as covariates in statistical testing to identify robust trends for both sexes.

### Study design.

IMPACC is a prospective longitudinal study designed to enroll 1,000 or more hospitalized patients with COVID-19 (23). The study cohort was enrolled from 20 hospitals affiliated with 15 geographically distributed academic institutions across the United States. Eligible participants included patients hospitalized with symptoms or signs consistent with COVID-19 and SARS-CoV-2 infection confirmed by reverse transcription PCR (RT-PCR). Detailed clinical assessments and nasal swabs, blood, and endotracheal aspirates (intubated patients only) were collected within 72 hours of hospitalization (visit 1) and on days 4, 7, 14, 21, and 28 after hospital admission for 1,152 participants, amounting to 3,077 sampling events. The levels of plasma proteomics (global and targeted; PPG and PPT, respectively), serum proteins (SPT), plasma metabolites (PMG), and nasal and PBMC mRNAs (NGX and PGX, respectively) were measured. The cohort was divided into training (*n* = 1,493) and test (*n* = 1,584) sets. The MCIA model was constructed from the preprocessed training multiomics data set, which was used on the test cohort for validation. The MCIA factors were identified and analyzed to comprehensively characterize the cross-tissue, multiomics immunological signatures that were associated with the severity and mortality. The measurements of nasal viral load and serum SARS-CoV-2 antibody titers as well as whole blood CyTOF for the same cohort were used to validate the immunological signatures and to develop further mechanistic insights into immune programs of COVID-19 severity and mortality. More details regarding the study cohort, sample processing, and batch correction can be found in the Supplemental Methods.

### Statistics.

Multiple comparisons were accounted for via Benjamini-Hochberg correction by default, with adj. *P* values of less than 0.05 considered significant unless otherwise stated. Linear mixed-effects modeling was used for differential analysis of baseline multiomics factors, selected pathway activities, and individual analytes across TG groups, after adjusting for sex and age as fixed effects and enrollment site as a random effect. Generalized mixed-effects modeling was utilized to investigate differential kinetics across conditions using samples from all visits after further adjusting for participant ID as a random effect. Unless otherwise specified, these sets of fixed and random effects for baseline and longitudinal samples were the default for all mixed-effects modeling. Linear mixed-effects regression was also adopted to generated interomics association *P* values using all visit samples. Models additionally adjusted for visit number and TG groups when investigating selected pathways and analytes from 2 assays used in MCIA factor construction to alleviate the strong coselection effects. High-contribution features for each assay were defined as the features whose absolute coefficients (from regressing features to factors) were 0.2 or greater and had adj. *P* values of significance (adj. *P* ≤ 0.01). Functional enrichment analysis for multiomics factors was performed using high-contribution features with a mHG test and a variety of publicly available databases, with only those with an adj. *P* value of less than 0.1 considered. The pathway activities were calculated as a weighted sum of a selected set of features in a pathway. See Supplemental Methods for detailed descriptions of all statistical analyses and models.

### Study approval.

NIAID staff conferred with the Department of Health and Human Services Office for Human Research Protections (OHRP) regarding the potential applicability of the public health surveillance exception (45CFR46.102) to the IMPACC study protocol. The OHRP concurred that the study satisfied the criteria for the public health surveillance exception, and the IMPACC study team sent the study protocol and the participant information sheet for review and assessment to the IRBs at the participating institutions. Twelve institutions elected to conduct the study as public health surveillance, whereas 3 sites with prior IRB-approved biobanking protocols elected to integrate and conduct the IMPACC study under their institutional protocols (The University of Texas at Austin, IRB 2020-04-0117; UCSF, IRB 20-30497; Case Western Reserve University, IRB STUDY20200573) with informed consent requirements. Participants enrolled under the public health surveillance exclusion were provided information sheets describing the study, samples to be collected, and plans for data deidentification and use. Those who opted not to participate after reviewing the information sheet were not enrolled. In addition, participants did not receive compensation for study participation while they were inpatients and were subsequently offered compensation during outpatient follow-ups.

### Data availability.

Data files are available at ImmPort Shared Data under accession number SDY1760 and in the NLM’s Database of Genotypes and Phenotypes (dbGaP) under accession number phs002686.v1.p1. All impacc-public-code analysis codes have been deposited in Bitbucket (https://bitbucket.org/kleinstein/impacc-public-code) and are publicly available. DOIs are included in Supplemental Table 11, which lists the key resources. Data required to re-create both the main and supplemental figures are included in the Supporting Data Values file.

## Author contributions

JPG, CM, RKP, the IMPACC Network, JDA, EM, KKS, GKF, PMB, LIRE, B Peters, B Pulendran, SHK, RPS, SF, LG, AO, EFR, EKH, MK, CBC, and NR conceived the study. JPG, CM, RKP, PS, AK, LX, AH, CPS, and LG conducted formal analysis. AK, RKP, CM, JPG, and LG designed software. LG designed the study methodology. SEB, SKS, FK, LBR, HVB, MW, HS, WLE, CRL, OL, MCA, HTM, DE, JPM, NDJ, GAM, and RRM provided resources. The IMPACC Network acquired funding. Supervision was provided by GKF, LIRE, B Peters, SF, SHK, and LG.

All authors wrote, edited, and reviewed the manuscript. The order of the co–first authors was determined alphabetically.

## Supplementary Material

Supplemental data

ICMJE disclosure forms

Supplemental table 1

Supplemental table 2

Supplemental table 3

Supplemental table 4

Supplemental table 5

Supplemental table 6

Supplemental table 7

Supplemental table 8

Supplemental table 9

Supporting data values

## Version 1 05/01/2024

Electronic publication
